# Generation of Long‐Lived Excitons in Room‐Temperature Phosphorescence 2D Organic and Inorganic Hybrid Perovskites for Ultrafast and Low Power‐Consumption Nonvolatile Photomemory

**DOI:** 10.1002/advs.202301028

**Published:** 2023-04-19

**Authors:** Jian‐Cheng Chen, Yu‐Dao Lu, Jung‐Yao Chen

**Affiliations:** ^1^ Department of Chemical Engineering National Chung Cheng University Chiayi 62102 Taiwan; ^2^ Department of Photonics National Cheng Kung University Tainan 70101 Taiwan; ^3^ Academy of Innovative Semiconductor and Sustainable Manufacturing National Cheng Kung University 70101 Tainan Taiwan

**Keywords:** block copolymer, low power consumption, nonvolatile photomemory, room‐temperature phosphorescence 2D organic‐inorganic hybrid perovskites, triplet exciton

## Abstract

Room‐temperature phosphorescence (RTP) two‐dimensional (2D) organic‐inorganic hybrid perovskites (OIHPs) that possess superior stability and efficient triplet energy transfer between inorganic parts and organic cations have been seen as promising materials in optoelectronic devices. However, the development of RTP 2D OIHP‐based photomemory has not been explored yet. In this work, the spatially addressable RTP 2D OIHPs‐based nonvolatile flash photomemory is first investigated to explore the function of triplet excitons in elevating the performance of photomemory. Thanks to the triplet excitons generated in RTP 2D OIHP, extremely low photo‐programming time of 0.7 ms, multilevel behavior of minimum 7 bits (128 levels), remarkable photoresponsivity of 19.10 AW^−1^ and significantly low power consumption of 6.79 × 10^−8^ J per bit can be achieved. The current study provides a new prospective in understanding triplet excitons function in nonvolatile photomemory.

## Graphical abstract

Benefitting from the triplet excitons generated in room‐temperature phosphorescence 2D organic‐inorganic hybrid perovskites, extremely low photo‐programming time of 0.7 ms, multilevel behavior of minimum 7 bits(128 levels), remarkable photoresponsivity of 19.10 AW^−1^ and significantly low power consumption of 6.79 × 10^−8^ J per bit can be achieved in the derived photomemory.

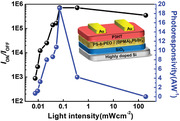

## 1 Introduction

Photonic integrated circuits (PICs) that integrate multiple photonic components have shown promising application on big data database with the features of high speed, large integration capacity, and low thermal effects. It is of great urgency to exploration of corresponding photonic components such as laser, optical waveguides, optical modulators, photodiodes, and nonvolatile flash photomemories. Among which, nonvolatile flash photomemories provide both photosensitive function and recording characteristics which could be further simplify the PICs design.^[^ ^1^, ^2^, ^3^, ^4^, ^5^ ^]^ Versatile photoactive materials have been executed in photomemory to enable the photo‐induced charge transfer between floating gate and charge‐transporting material.^[^ ^1^, ^2^, ^6^, ^7^, ^8^ ^]^ When considering that the mechanism of organic photovoltaics is similar to nonvolatile flash photomemories, sufficient exciton lifetime seem to be required condition for effective charge separation for high‐performance optoelectronic devices.^[^ ^5^, ^7^ ^]^

Phosphorescence is the long‐life emission which involves the flip of electron spin state from singlet to triplet excited state through the intersystem crossing and then relaxes back to the ground state via radiative relaxation. Recently, room‐temperature phosphorescence (RTP) has received considerable attention due to its important applications in anti‐counterfeiting technologies, photodynamic therapy, bioimaging, and light‐emitting devices.^[^ ^9^, ^10^ ^]^ Crystallization‐induced phosphorescence(CIP), metal‐organic frameworks (MOFs), and two‐dimensional (2D) organic‐inorganic hybrid perovskites (OIHPs) have been developed to enable RTP.^[^ ^10^, ^11^, ^12^, ^13^, ^14^ ^]^ Compared to CIP and MOFs which restrict the intramolecular motion by ordered structure, 2D OIHPs with triplet‐triplet Dexter energy transfer (TTDET) between inorganic parts and organic conjugated cations provide different platforms to generate RTP.^[^ ^15^, ^16^ ^]^ Due to the effective energy transfer between inorganic Wannier excitons to organic Frenkel excitons, 2D OHIPs possess high quantum yield of ≈100% compared to the intersystem crossing(ISC) in pure organic materials.^[^ ^12^, ^17^, ^18^ ^]^ Therefore, RTP 2D OIHPs with high ambient stability, superior charge‐carrier mobility, and microsecond or millisecond‐scale exciton lifetime may be a promising photoactive material in photomemories to achieve superior photo‐responsive performance. However, until now, engineering the exciton lifetime of photoactive materials on the performance of photomemories is still limited.^[^ ^19^, ^20^, ^21^, ^22^, ^23^ ^]^

Block copolymers (BCPs) are widely known for the self‐assembled structure which can be engineered by block ratio, solvent annealing, and thermal annealing.^[^ ^24^, ^25^, ^26^ ^]^ Besides, the supramolecular self‐assembled of BCPs with functional materials enables the exploitation of the morphology of composite film on elevating the performance of derived optoelectronic devices, especially for floating‐gate nonvolatile memory.^[^ ^27^, ^28^, ^29^, ^30^, ^31^, ^32^, ^33^, ^34^ ^]^ In addition, solvent vapor annealing (SVA) not only swells the polymer chains but also provides an effective method to increase grain size and the crystallinity of OIHPs embedded in BCP/OIHPs.^[^ ^35^, ^36^, ^37^, ^38^, ^39^ ^]^

**Figure 1:**
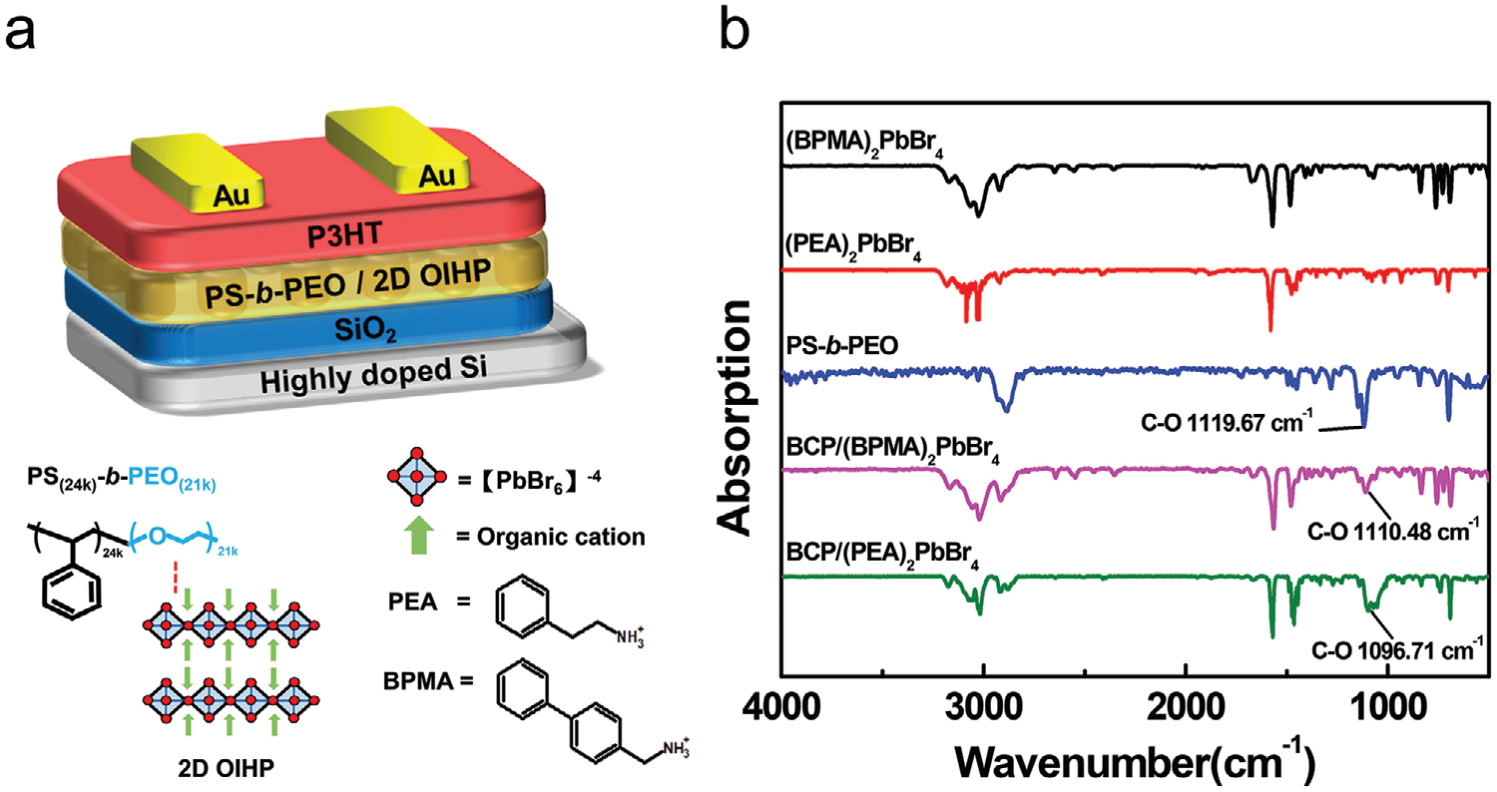
a) Device configuration of the BCP/2D OIHP‐based photomemory and chemical structure of materials. b) FTIR absorption spectra of 2D OIHP, BCP, and BCP/2D OIHP.

Herein, to exploit the effect of exciton lifetime on the performance of photomemories, two 2D OIHPs 2‐phenylethanaminium lead bromide ((PEA)_2_PbBr_4_) and 4‐biphenylmethylammonium lead bromide ((BPMA)_2_PbBr_4_) are spatially confined in polystyrene‐*block*‐poly(ethylene oxide) (PS‐*b*‐PEO) to serve as the photoactive charge‐trapping materials with *p*‐type semiconductor poly(3‐hexylthiophene‐2,5‐diyl) (P3HT) as the charge‐transporting channel in nonvolatile floating‐gate photomemory (**Figure** **1**a). (BPMA)_2_PbBr_4_ with 4‐biphenylmethylammonium as the organic cation has been shown to possess RTP owing to the longer conjugated cation which enables the energy transfer between inorganic [PbBr_6_]^−4^ and organic 4‐biphenylmethylammonium through TTDET.^[^ ^18^ ^]^ In contrast, (PEA)_2_PbBr_4_ with shorter conjugated length and accordingly higher triplet exciton energy inhibits the energy transfer; therefore, only fluorescence emission can be observed. Through triggering the energy transfer between inorganic parts and organic cations by extending the conjugated length of organic cations, ultrafast and low energy consumption RTP 2D OIHPs based photomemory is first demonstrated. Results of this study may provide the guideline for the design of high‐performance photomemory toward the generation of long‐lived excitons.

## 2 Results

### 2.1 Morphological Analysis of BCP/2D OIHP Composite Film

For investigating the interaction between PS‐*b*‐PEO(BCP) and 2D OHIPs in BCP/2D OHIP composite film, Fourier transform infrared spectroscopy (FTIR) analysis was executed (Figure 1b). The absorption peak at 1119.67 cm^−1^ specified as the C—O bond stretching from PEO moiety in BCP is shifted to 1110.48 cm^−1^ in the BCP/(BPMA)_2_PbBr_4_ composite film. Meanwhile, the similar peak shift of C–O bond stretching also can be identified in the BCP/(PEA)_2_PbBr_4_ composite film. The shift reveals that the Lewis acid‐base interaction can be contributed to the precursor Pb^2+^ ion coordinated with the oxygen in PEO moiety. In our previous work, the strong chelating effect between Pb^2+^ ion and oxygen achieves the excellent anti‐solvent feature of PS‐*b*‐PEO/2D OIHP composite film that enable solution processable P3HT as a charge‐transporting channel.^[^ ^1^, ^4^, ^40^ ^]^ Figure S1a,b, Supporting Information, presents the transmission electron microscopy (TEM) imagines of the BCP/(PEA)_2_PbBr_4_ and BCP/(BPMA)_2_PbBr_4_ composite films, respectively. The dark regions can be assigned to the PEO/2D OHIP domains which leads the PS cylinders well‐distributed in both BCP/2D OHIP composite films. Grazing‐incidence small‐angle scattering (GISAXS) was executed to confirm self‐assembling microphase separation in BCP/2D OHIP composite films as shown in Figure S1c and S1d, Supporting Information. The distinguishable scattering vector (*q*) located at 0.0154 Å^−1^ and 0.0132 Å^−1^ corresponding to domain spacing(*d*) of 40.7 nm and 47.5 nm for the BCP/(PEA)_2_PbBr_4_ and BCP/(BPMA)_2_PbBr_4_ composite films, respectively. At a fixed molar ratio between PEO moiety and 2D OIHPs, the relative larger domain spacing may result from the bigger molecular size of 4‐biphenyl methyl ammonium compared to 2‐phenylethanaminium ammonium. 2D Diffraction patterns (Figure S1e,f, supporting information) from grazing‐incidence wide‐angle scattering (GIWAXS) confirm the formation of 2D OIHPs in composite films. In the out‐of‐plane direction, the lamella packing (001) from the adjacent inorganic layers leads to the scattering vector at 0.38 Å^−1^ and 0.26 Å^−1^ with the spacing of 16.5 Å and 24.2 Å for the BCP/(PEA)_2_PbBr_4_ and BCP/(BPMA)_2_PbBr_4_ composite films, respectively. The relative thicker lamella packing may stem from the larger 4‐biphenyl methyl ammonium compared to 2‐phenylethanaminium.

### 2.2 Charge Transfer Behavior in BCP/2D OIHP‐based Photomemory

**Figure 2:**
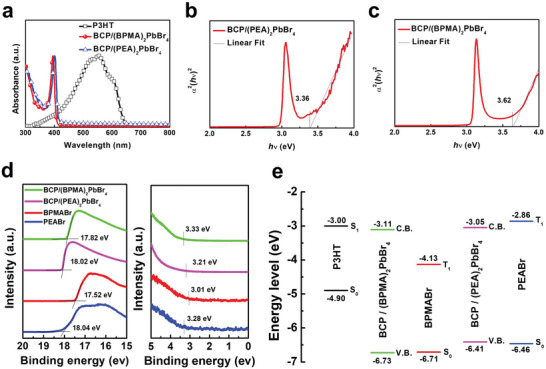
a) Optical absorption of the BCP/2D OIHP composite films and P3HT film. tau plot of b) BCP/(PEA)_2_PbBr_4_ and c) BCP/(BPMA)_2_PbBr_4_. d) UPS spectra of BCP/(PEA)_2_PbBr_4_, BCP/(BPMA)_2_PbBr_4_, PEABr, and BPMABr. e) Energy level diagram of P3HT, BCP/(PEA)_2_PbBr_4_, BCP/(BPMA)_2_PbBr_4_, PEABr and BPMABr.

To confirm optical properties of the composite films, UV–Visible absorption spectra and steady‐state photoluminescence spectrum (PL) were further explored. UV–Visible absorption spectra of the BCP/(PEA)_2_PbBr_4_, BCP/(BPMA)_2_PbBr_4_ composite films, and P3HT film are shown in **Figure** **2**a. Due to the quantum confinement within the 2D inorganic lattice, the sharp excitonic absorption peaks ≈402 nm for both BCP/(PEA)_2_PbBr_4_ and BCP/(BPMA)_2_PbBr_4_ composite films can be observed which proves the formation of 2D OIHP crystallites in composite film. For P3HT thin film, the absorption spectrum is in the range from 300 nm to 800 nm which enable light recording behavior in whole visible light spectrum for the derived photomemories. From the optical absorption (Figure 2b,c), the bandgap is ≈3.36 eV and 3.62 eV for BCP/(PEA)_2_PbBr_4_ and BCP/(BPMA)_2_PbBr_4_, respectively. From ultraviolet photoelectron spectroscopy (UPS) results (Figure 2d), the estimated valence band maximum (VBM) of BCP/(PEA)_2_PbBr_4_, BCP/(BPMA)_2_PbBr_4_, PEABr, and BPMABr are −6.41 eV, −6.73 eV, ‐6.46 Ev, and −6.71 eV, respectively. Combining these values with the corresponding optical band gaps enables the determination of the estimated conduction band minimum (CBM) to be −3.05 eV and −3.11 eV for BCP/(PEA)_2_PbBr_4_ and BCP/(BPMA)_2_PbBr_4_, respectively. The energy levels of 2D OIHP composite films and P3HT film are summarized in Figure 2e. Both the BCP/2D OIHP composite films possess lower VBM and CBM than those of P3HT thin film ensuring that the photo‐induced charge transfer between P3HT and 2D OIHP. When considering the energy difference between the excited triplet exciton of corner‐sharing [PbBr_6_]^4−^ octahedra (3.06 eV) and PEA (3.6 eV),^[^ ^41^ ^]^ the higher exciton energy of organic cation inhibits the TTDET from inorganic part to organic part, and thus only fluorescence can be observed in PL spectrum. In contrast, BPMA consists of two connected phenyl groups with the excited triplet state (*T*_1_) of ≈2.58 eV which is smaller than the excited triplet state of inorganic layer and accordingly enables theTTDET between inorganic layer and organic cation.^[^ ^18^, ^41^ ^]^ It has been reported that the Dexter energy transfer rate is greatly faster than the charge transfer rate. This indicates that photo‐induced excitons in the inorganic well will transfer to the triplet state of organic cations at first, and then triplet excitons implement the holes transfer process from the highest occupied molecular orbital (HOMO) of organic cations to the HOMO of P3HT. Therefore, HOMO energy level of 2D OIHP precursor PEABr and BPMABr were probed by UPS as well. From UPS spectra (Figure 2d), the estimated HOMO energy level is −6.46 eV and −6.71 eV for PEABr and BPMABr, respectively. Based on the excited triplet state and HOMO energy level of PEA and BPMA, the T_1_ energy level would be estimated as −2.86 eV and −4.13 eV, respectively. It should be noted that the lower T_1_ energy level of BPMA also retard the electron transfer from BPMA to the LUMO of P3HT which enables the photo‐recordable functionality as discussed in the following.

**Figure 3:**
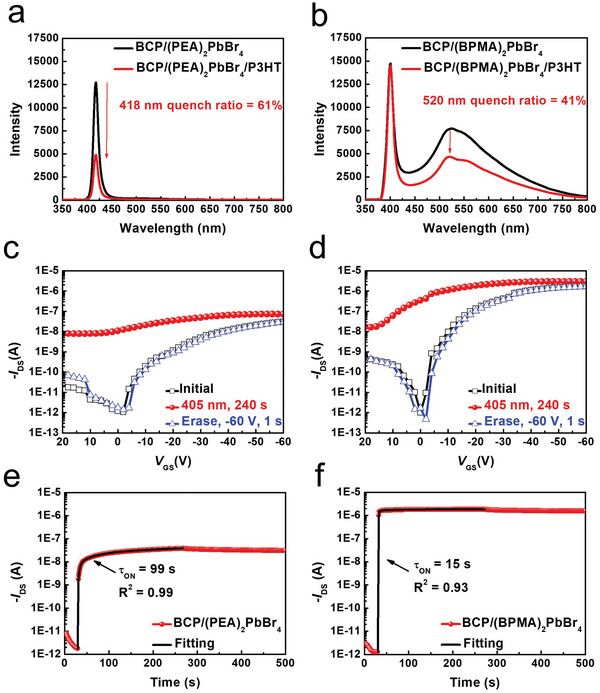
Photoluminescence of the composite films on quartz and on P3HT with excitation at 375 nm: a) BCP/(PEA)_2_PbBr_4_ and BCP/(PEA)_2_PbBr_4_/P3HT b) BCP/(BPMA)_2_PbBr_4_ and BCP/(BPMA)_2_PbBr_4_ /P3HT. Transfer characteristic of c) BCP/(PEA)_2_PbBr_4_and d) BCP/(BPMA)_2_PbBr_4_ based photomemory at *V*_DS_ = −60 V (405 nm, 50 mW cm^−2^). The photo‐responsive current toward time under violet‐light illumination (405 nm, 50 mW cm^−2^) at *V*_DS_ = −60 V of e) BCP/(PEA)_2_PbBr_4_ and f) BCP/(BPMA)_2_PbBr_4_.

To investigate the charge transfer process between BCP/2D OIHP composite films and P3HT thin film, photoluminescence (PL) quenching experiment was employed. **Figure** **3**a,b shows the PL spectra of the BCP/(PEA)_2_PbBr_4_ and BCP/(BPMA)_2_PbBr_4_ composite films with and without P3HT film, respectively. Under 375 nm laser excitation, both BCP/(PEA)_2_PbBr_4_ and BCP/(BPMA)_2_PbBr_4_ composite films have a characteristic peak from the relaxation of inorganic excitons directly to the ground states leading to distinct fluorescence at ≈410 nm. In contrast, the BCP/(BPMA)_2_PbBr_4_ demonstrates characteristic phosphorescence emission covered from 500 nm to 700 nm due to the TTDET between the inorganic layer and organic cation. To further confirm the phosphorescence feature of (BPMA)_2_PbBr_4_, the optical properties of pure (BPMA)_2_PbBr_4_ film was also executed as shown in Figure S2, Supporting Information. In the static PL spectrum of (BPMA)_2_PbBr_4_ film (Figure S2a, Supporting Information), the broad emission peaks can be roughly deconvoluted into three peaks located at 520 nm, 560 nm, and 610 nm which are similar to the characteristic phosphorescence emission of BPMABr molecule typically observed in low temperature (77K) as shown in Figure S2b, Supporting Information. Besides, the transient decay curve of (BPMA)_2_PbBr_4_ film at the phosphorescence emission of 530 nm displays the exciton lifetime of 0.73 ms (Figure S2c, Supporting Information). Compared to the nanosecond‐scale exciton lifetime like the one of (PEA)_2_PbBr_4_, a longer exciton lifetime may lead to a longer exciton diffusion length. Therefore, the BCP/(BPMA)_2_PbBr_4_ with energy transfer between inorganic Wannier excitons to organic Frenkel excitons may be a promising photoactive material to optimize the optoelectronic properties of derived photomemory. Since PL quenching is a measure of exciton separation at the donor‐acceptor interface, PL quenching at the specific wavelength region also reveals the charge transfer behavior in singlet excitons and triplet excitons. Both BCP/2D OIHP composite films show PL quenching in conjunction with P3HT film which confirm hole transfer from 2D OIHP to P3HT, however, with different mechanisms in two systems. For (PEA)_2_PbBr_4_ system, the composite film with P3HT is quenched by 61% with respect to the PL intensity of composite films without P3HT. On the other hand, it is interesting to noted that the PL quenching only appeared at the phosphorescence peak of 520 nm with a quenching ratio of 41% while the PL signal from the relaxation of inorganic excitons almost keeps the same intensity in conjunction with P3HT. It is worth mentioning that several research works have shown that the Dexter energy transfer rate is ≈10 ns^−1^ which is faster than the charge transfer rate (≈10^−2^ ns^−1^).^[^ ^1^, ^4^, ^17^, ^42^ ^]^ Therefore, the above inequal PL quenching in BCP/(BPMA)_2_PbBr_4_ system can be inferred that the fast energy transfer from the inorganic excitons to organic exciton inhibits the hole transfer from [PbBr_6_]^−4^ to P3HT while long‐lived triplet excitons of BPMA in BCP/(BPMA)_2_PbBr_4_ provide the sufficient exciton lifetime and consequently dominate the hole transfer process.

Time‐resolved PL (TRPL) spectra were measured to confirm the charge transfer behavior between inorganic excitons in BCP/(BPMA)_2_PbBr_4_ composite films and P3HT. Figure S3a,b (Supporting Information) are 2D contour plots of the TRPL spectra of the BCP/(BPMA)_2_PbBr_4_ and BCP/(BPMA)_2_PbBr_4_/P3HT, respectively. The corresponding fluorescence decay curves at the wavelength of 402 nm from the relaxation of inorganic excitons are shown in Figure S3c (Supporting Information). The transient PL decay could be described by using the biexponential fitting curve. The average lifetime *τ*_PL_ of BCP/(BPMA)_2_PbBr_4_ and BCP/(BPMA)_2_PbBr_4_/P3HT is 0.10 ns ad 0.07 ns, respectively. The barely distinguishable difference in *τ*_PL_ of the inorganic exciton further confirms that inorganic [PbBr_6_]^−4^ excitons do not take part in the charge transfer process. To investigate the charge transfer behavior of triplet exciton, transient behavior of phosphorescence emission at 520 nm was also probed as shown in Figure S4, Supporting Information. By fitting the curve with triexponential decay, the average triplet exciton lifetime of BCP/(BPMA)_2_PbBr_4_ obviously drops from 228.60 µs to 94.60 µs when contacting with P3HT thin film. All TRPL results are summarized in Table S1, Supporting Information. The significant difference in kinetic behavior of singlet excitons and triplet excitons highlights the pivotal role of long‐lived exction lifetime in the charge transfer process between (BPMA)_2_PbBr_4_ and P3HT.

### 2.3 Performance of BCP/2D OIHP Composite Film Based Photomemory

The transfer curves were measured by sweeping gate voltage (*V*_GS_) from 20 V to −60 V under fixed drain voltage (*V*_DS_) of −60 V to investigate the photo‐responsive properties of BCP/2D OIHP composite film‐based photomemories. As shown in Figure 3c,d for BCP/(PEA)_2_PbBr_4_ and BCP/(BPMA)_2_PbBr_4_ based photomemories, respectively, apparent threshold voltage shifts after light illumination (405 nm, 50 mW cm^−2^) for 240 s can be observed, leading to current switching ratios of 1.02 × 10^4^ and 3.46 × 10^5^ for BCP/(PEA)_2_PbBr_4_ and BCP/(BPMA)_2_PbBr_4_ based photomemories, respectively, at *V*_GS_ = 0 V. It should be noted that the charge‐carrier mobility in saturation‐regime is 8.08 × 10^−5^ and 2.02 × 10^−3^ cm^2^V s^−1^ for BCP/(PEA)_2_PbBr_4_ and BCP/(BPMA)_2_PbBr_4_ based photomemory, respectively. The difference in charge‐carrier mobility might stem from the higher surface roughness of P3HT thin film on BCP/(PEA)_2_PbBr_4_ composite film with the root‐mean‐square roughness value (*R*q) of 5.19 nm compared to the P3HT thin film on BCP/(BPMA)_2_PbBr_4_ (*R*q of 2.83 nm) as shown in Figure S5 (Supporting Information), leading to the severe charge‐carrier scattering. The electrical‐erasing through applying a gate bias at −60 V for 1s can be further employed to restore the studied device to their initial state. To compare the dynamics of photo‐induced electron transfer process in two BCP/2D OIHP systems, the temporal responses of the studied devices under light illumination (405 nm, 50 mW cm^−2^) were probed by recording the *I*_DS_ at a fixed *V*_DS_ = −60 V and *V*_GS_ = 0 V and are shown at Figure 3e,f for BCP/(PEA)_2_PbBr_4_ and BCP/(BPMA)_2_PbBr_4_, respectively. The rising parts can be described by Equation 1.

(1) $$I_{\mathrm{DS}}=A\left(1-\exp\left(\frac{-t}{\tau_{\mathrm{ON}}}\right)\right)+I_{\mathrm{dark}}$$

where *t* is time, *τ*_ON_ is the characteristic ON time, *I*_dark_ is the dark current, and *A* is the scaling constant. The *τ*_ON_ is 99 s and 15 s for BCP/(PEA)_2_PbBr_4_ and BCP/(BPMA)_2_PbBr_4_ derived photomemories, respectively. The superior ON/OFF current ratio and smaller *τ*_ON_ in BCP/(BPMA)_2_PbBr_4_‐based photomemory are believed to stem from the long‐lived triplet exciton from BPMA.

**Figure 4:**
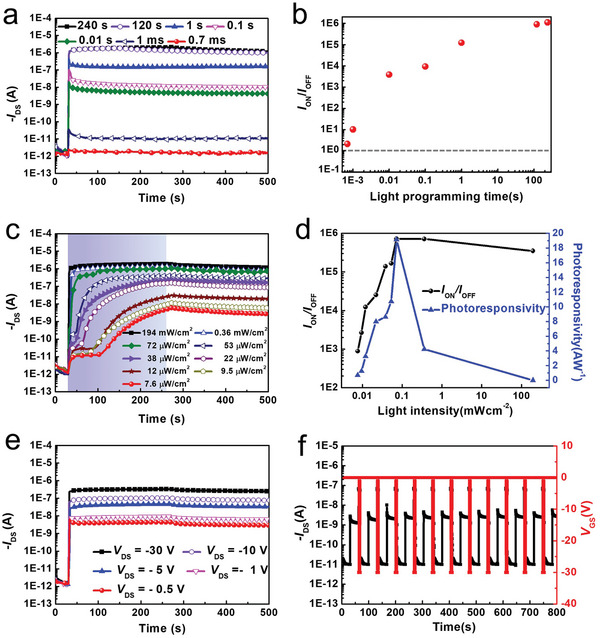
a) Temporal *I*_DS_ curves of the BCP/(BPMA)_2_PbBr_4_ based‐photomemory at *V*_DS_ = −60 V with different illuminating time ranging from 0.7 ms to 240 s (at 405 nm, 194 mW cm^−2^). b) *I*_ON_/*I*_OFF_ ratio toward light programming time diagram. c) Temporal *I*_DS_ curves of the BCP/(BPMA)_2_PbBr_4_ based‐photomemory at *V*_DS_ = −60 V with varying illumination intensities from 7.6 µWcm^−2^ to 194 mWcm^−2^ (the blue shadow indicates the illumination period). d) *I*_ON_/*I*_OFF_ ratio and photoresponsivity toward light intensity diagram. e) Temporal *I*_DS_ curves of the BCP/(BPMA)_2_PbBr_4_ based‐photomemory at *V*_DS_ ranged from −0.5 V to −60 V with light programming (405 nm, 194 mWcm^−2^) for 240 s. (f) WRER switching behavior of the BCP /(BPMA)_2_PbBr_4_‐based photomemory was measured at a fixed *V*_DS_ = −0.5 V and programmed by light illumination (405 nm, 194 mWcm^−2^) for 1s and erased at *V*_GS_ = −30 V for 10 s.

**Figure 5:**
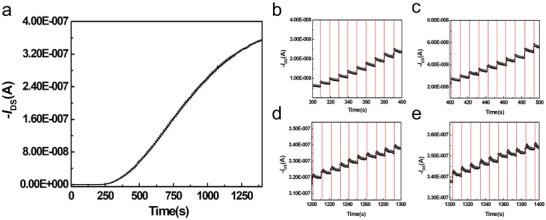
Measurement of BCP/(BPMA)_2_PbBr_4_/P3HT device multilevel behavior at *V*_DS_ = −60 V with an illuminating time 0.7 ms laser pulses (at 405 nm, 194 mWcm^−2^) every 10 s. The ability of a multilevel memory behavior for: a) 0–1400 s b) 300–400 s c) 400–500 s d) 1200–1300 s e) 1300–1400 s.

To further explore the effect of generated triplet exciton on the charge transfer between BCP/(BPMA)_2_PbBr_4_ and P3HT, various programming time were measured ranged from 0.7 ms to 240 s with 405 nm violet‐light and intensity of 194 mW cm^−2^ (**Figure** **4**a). As shown in Figure S6 (Supporting Information), the enlarged image demonstrates the photocurrent of BCP/(BPMA)_2_PbBr_4_‐based photomemory still showed decent ON/OFF current ratio of 2.4 upon an extremely short illumination duration of 0.7 ms. All the ON/OFF current ratio under different illumination time are summarized in Figure 4b. Benefitting from the long exciton lifetime of BCP/(BPMA)_2_PbBr_4_‐based photomemory, light‐intensity distinguishable behavior with light intensity ranging from 7.6 µW cm^−2^ to 194 mW cm^−2^ can be realized (Figure 4c). As summarized in Figure 4d, the maximum photoresponsivity of 19.10 AW^−1^ can be achieved. Compared to the state‐of‐art perovskite‐based photomemories (Table S2, Supporting Information),^[^ ^19^, ^20^, ^21^, ^22^, ^23^ ^]^ BCP/(BPMA)_2_PbBr_4_‐based photomemory show the lowest photo‐recordable time and the highest photoresponsivity which may stem from the fact that the generation of triplet excitons enable enough exciton diffusion length for charge transfer process. As the lateral driving voltage decreased to ‐0.5 V, the BCP/(BPMA)_2_PbBr_4_‐based photomemory still display a superior ON/OFF current ratio of 2.12 × 10^3^ owing to its high photoresponsivity (Figure 4e). Consecutive optical writing‐reading‐electrical erasing‐reading (WRER) test was probed to examine the switching behavior of BCP/(BPMA)_2_PbBr_4_‐based photomemory, as portrayed in Figure 4f where cycle test consists of photo‐gating (405 nm, 194 mW cm^−2^) for 1 s, reading process at *V*_DS_ of −0.5 V, electrical‐erasing (*V*_GS_ of −30 V for 10 s) and then reading process at *V*_DS_ of −0.5 V. To ensure the reliable endurance, WRER for 30 cycles also demonstrate in Figure S7, Supporting Information. The stable and distinguishable ON and OFF currents manifests the low power consumption of the studied device for the practical application in the future. Owing to the extremely short photo‐programming time, the BCP/(BPMA)_2_PbBr_4_‐based photomemory can realize the multilevel behavior, manipulated by consecutively giving a 405 nm violet light (194 mW cm^−2^, 0.7 ms) for every 10 s at fixed *V*_DS_ = −60 V (**Figure** **5**a). By zooming the different time intervals of the photocurrent in Figure 5b–e, 7 bit (128 levels) per cell can be achieved with an extremely low power consumption of 6.79 × 10^−8^ J per bit.

### 2.4 Device Mechanism of BCP/2D OIHP Composite Film Derived Photomemory

**Figure 6:**
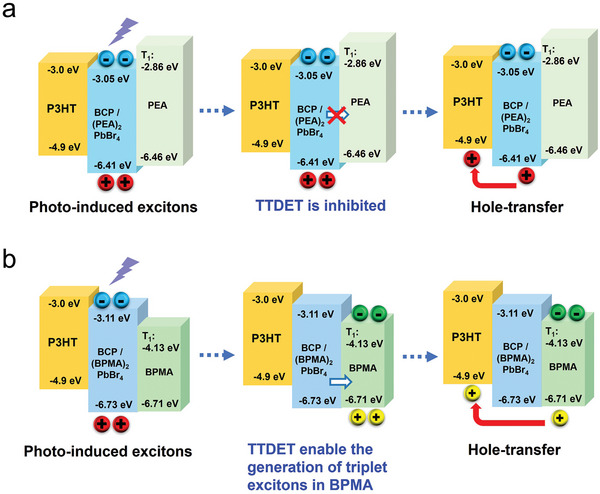
The schematic diagram of operating mechanism of a) BCP/(PEA)_2_PbBr_4_ and b) BCP/(BPMA)_2_PbBr_4_ derived photomemory.

Based on the results, we propose the possible operating mechanisms of BCP/2D OIHP‐based photomemory as illustrated in **Figure** **6**. For BCP/(PEA)_2_PbBr_4_ derived photomemory (Figure 6a), due to the high triplet exciton energy of PEA (3.6 eV) compared to the triplet exciton of corner‐sharing [PbBr_6_]^4−^ octahedra (3.06 eV), the TTDET between inorganic [PbBr_6_]^−4^ and organic layer is inhibited. Therefore, only photo‐induced excitons in inorganic layer participate in the hole transfer process. In contrast, for BCP/(BPMA)_2_PbBr_4_ derived photomemory as shown in Figure 6b, the small triplet exciton energy of BPMA enable the TTDET between inorganic [PbBr_6_]^−4^ and organic layer under light illumination and consequently the generation of organic triplet excitons. The highly efficient and fast energy transfer rate between [PbBr_6_]^−4^ and organic layer retards the occurrence of hole transfer between inorganic [PbBr_6_]^−4^ and P3HT. Since the exciton diffusion length is influenced by both the lifetime and diffusivity, the generation of long‐lived triplet excitons may inhibit the geminate recombination and enable adequate exciton diffusion length to promote the following exciton separation process and accordingly realize the superior photo‐responsive performance.

## 3 Conclusion

In summary, our work first demonstrates the nonvolatile photomemory with BCP/RTP 2D OIHP as charge‐trapping material and *p*‐type semiconductor P3HT as the channel to elucidate the effect of generated triplet excitons on the optoelectronic characteristics of photomemory. Thanks to the long exciton lifetime of triplet excitons generated in RTP 2D OIHP, low photo‐programming time of 0.7 ms, photoresponsivity of 19.10 AW^−1^ and low power consumption of 6.79 × 10^−8^ J per bit can be achieved. These results reveal the peculiar charge transfer mechanism in RTP 2D OIHP‐based nonvolatile photomemory and address triplet excitons in elevating the performance of nonvolatile photomemory.

## 4 Experimental Section

### Materials

Lead(II) bromide (PbBr_2_) (99.999% trace metals basis), N, N‐dimethylformamide (anhydrous, 99.8%), and toluene (anhydrous, 99.8%) were purchased from Sigma‐Aldrich. 2‐phenylethanaminium bromide (PEABr) (powder) was purchased from Greatcell Solar Materials. 4‐biphenylmethylaminium bromide (BPMABr) (powder) was purchased from Lumtec. Poly(3‐hexylthiophene) (P3HT) (Mw ≈ 50 000, 90%–95% regioregular) was obtained from Rieke Metals Inc. Poly(styrene)‐*b*‐poly(ethylene oxide)(PS‐*b*‐PEO) block copolymers (Mn(PS) = 24 000 gmol^−1^, Mn(PEO) = 21 000 g mol^−1^; Mw/Mn = 1.09) was purchased from Polymer Source, Inc., Canada.

### Fabrication of Photomemory

The highly doped n‐type silicon wafer with 100 nm‐thick SiO_2_ was cleaned by an ultrasonic cleaner in toluene, acetone, isopropyl alcohol before being used as gate and dielectric for photomemory. First, the 10 mg mL^−1^ PS‐*b*‐PEO and 0.05 M 2D OIHP hybrid solutions in DMF were spin‐cast at spin rate of 1000 rpm for 60 s on wafer and then solvent annealed under saturated ethanol vapor for 16 h in N_2_‐filled glove box at room temperature. The measured capacitance of the PS‐*b*‐PEO/2D OHIP/SiO_2_ as the dielectric layer is ≈ 19.8 nF cm^−2^ at 10 kHz. Then 1 mg mL^−1^ P3HT solution in toluene was spin‐cast onto the PS‐*b*‐PEO/2D OIHP composite film. Subsequently, top‐contact and bottom‐gate configuration with a channel length (L) and width (W) of 50 µm and 1000 µm, respectively was defined by 50 nm‐thick thermal evaporated gold electrodes.

### Characterization

The interaction between PS‐*b*‐PEO and 2D OIHP was analyzed using Fourier transform infrared (FTIR, Miracle ATR, Perkin Elmer). Transmission electron microscopy (TEM, JEM‐2100F Electron Microscope) were used to investigate the distribution of 2D OIHP in the composite film. The UV‐Vis spectra were characterized using Shimadzu UV‐2600 spectrometer. The ultraviolet photoelectron spectroscopy (UPS, ULVAC‐PHI PHI 5000 Versaprobe II) with a photon energy of 21.22 eV was used to measure the valence band maxima of BCP/2D OIHP composite film. The photoluminescence (PL) and time‐resolved photoluminescence (TRPL) spectra for probing singlet exciton lifetime were measured using 375 nm excitation wavelength and collected using a fiber, coupled with Hamamatsu C10910 streak camera and M10913 slow single‐sweep unit at the NSRRC, Taiwan. TRPL spectra for probing triplet exciton lifetime were collected using a photomultiplier (PMA‐C‐192‐N‐M, PicoQuant) and analyzed by a time‐correlated single‐photon counting board (TimeHarp 260, PicoQuant). The sample was excited by a 375 nm picosecond pulsed laser diode (LDH‐IB‐375‐B, PicoQuant) triggered by laser driver (Taiko PDL M1, PicoQuant) with a repetition rate of 80 MHz under burst mode.

Grazing incident small‐angle X‐ray scattering (GISAXS) at Taiwan Light Source (TLS) beamline 23A and grazing incident wide‐angle X‐ray scattering (GIWAXS) at TLS beamline 25A in the NSRRC, Taiwan were executed to find out the microphase separation in BCP/2D OIHP and ordered planar structure of 2D OIHP, respectively. The photo‐responsive properties of photomemory were probed by using Keithley 4200‐SCS semiconductor parameter analyzer in a N_2_‐filled glove box at room temperature with 405 nm laser. The laser pulse width and intensity were manipulted using Keithley 2636. Photoresponsivity was determined through the equation (*I*_ON_‐*I*_OFF_)/light intensity/illuminated area. Power consumption per bit was determined through the equation illumination intensity × illuminating time × illuminated area.

## Conflict of Interest

The authors declare no conflict of interest.

## Supporting information

Supporting information

## Data Availability

The data that support the findings of this study are available from the corresponding author upon reasonable request.
