# Supplementary material for: Generation of Long‐Lived Excitons in Room‐Temperature Phosphorescence 2D Organic and Inorganic Hybrid Perovskites for Ultrafast and Low Power‐Consumption Nonvolatile Photomemory
Source: Adv Sci (Weinh). 2023 Apr 19;10(19):2301028. doi: 10.1002/advs.202301028 (PMC10323654; doi:10.1002/advs.202301028)
Supplement: Supplementary file 1 — Supporting information [file ADVS-10-2301028-s001.pdf]

ADVANCED SCIENCE
Open Access

Supporting Information

for *Adv. Sci.*, DOI 10.1002/advs.202301028

Generation of Long-Lived Excitons in Room-Temperature Phosphorescence 2D Organic and Inorganic Hybrid Perovskites for Ultrafast and Low Power-Consumption Nonvolatile Photomemory

*Jian-Cheng Chen, Yu-Dao Lu and Jung-Yao Chen**

Generation of Long-lived Excitons in Room-temperature Phosphorescence 2D Organic and Inorganic Hybrid Perovskites for Ultrafast and Low Power-consumption Non-volatile Photomemory

Jian-Cheng Chen, [a] *Yu-Dao Lu,* [b] *Jung-Yao Chen,* [a,b,c*]

Table S1. TRPL fitting results of BCP/$(BPMA)_2PbBr_4$ and BCP/$(BPMA)_2PbBr_4$/P3HT.

Sample	τ_{PL} at 402 nm (ns)	τ_{PL} at 520 nm (μs)
BCP/$(BPMA)_2PbBr_4$	0.10	228.6
BCP/$(BPMA)_2PbBr_4$/P3HT	0.07	95.6

Table S2. The state-of-art perovskite based photomemories and the corresponding electrical performance.

**Device structure**	**Light wavelength (nm)**	**Light intensity (mW cm^{-2})**	**Lowest Photo-recordable time (s)**	**Maximum photoresponsivity (mA W^{-1})**	**Reference**
Si/SiO_2/PS/ $CH_3NH_3PbBr_3$/ pentacene/Au	450	71	30	5.63×10^{-2}	*Adv. Mater.* **2017,** 29, 1702217
Si/SiO_2/PMMA/ $CsPbBr_3$ quantum dots/pentacene/Au	365	0.153	2	~1.31×10^{3}	*Adv. Mater.* **2018**, 30, 1802883.
Si/SiO_2/ PMMA/ $CH_3NH_3PbBr_3$ /P3HT nanofiber/Au	450	71	120	3.13×10^{2}	*Nanoscale,* **2018,** 10, 18869.
Si/SiO_2/P2VP/CH_3NH_3/ pentacene/Au	405	48.5	5	~4.12×10^{-2}	*Adv.Electron. Mater.* **2020**, 6, 2000458
Si/SiO_2/PS-*b*-PEO/ $MAPbBr_3$/P3HT/Au	520	454.36	5×10^{-3}	1.31×10^{2}	*Adv. Funct. Mater.* **2020**, 30, 2000764.
Si/SiO_2/$CH_3NH_3PbBr_3$ /P2VP/BPE-PTCDI/Au	530	10	60	1.9×10^{-3}	*ACS Appl. Mater. Interfaces* **2021,** 13, 20417.
Si/SiO_2/$FAPbBr_3$ quantum dot/P3HT/Au	450	623.23	1×10^{-3}	6.1	*Adv. Funct. Mater.* **2021**, 31, 2105911.
Si/SiO_2/$CH_3NH_3PbBr_3$ quantum dots/P3HT nanofiber/Au	405	42.5	1	2.17	*Adv.Mater. Technol.***2021,** 6, 2100080
Si/SiO_2/$CsPbBr_3$ quantum dots/P3HT/Au	405	42.5	1	6.91	*Adv. Funct. Mater.***2022,** 32, 2107925
Si/SiO_2/PS-b-PEO/ $FAPbBr_3$–5%PEABr/P3HT/Au	450	0.85	5×10^{-3}	9.20×10^{2}	*Adv. Funct. Mater.* **2022,** 32, 2112521.

Si/SiO_2/PS-b-PEO/ $(BPMA)_2PbBr_4$/P3HT/Au	405	194	7×10^{-4}	1.91×10^{4}	This work

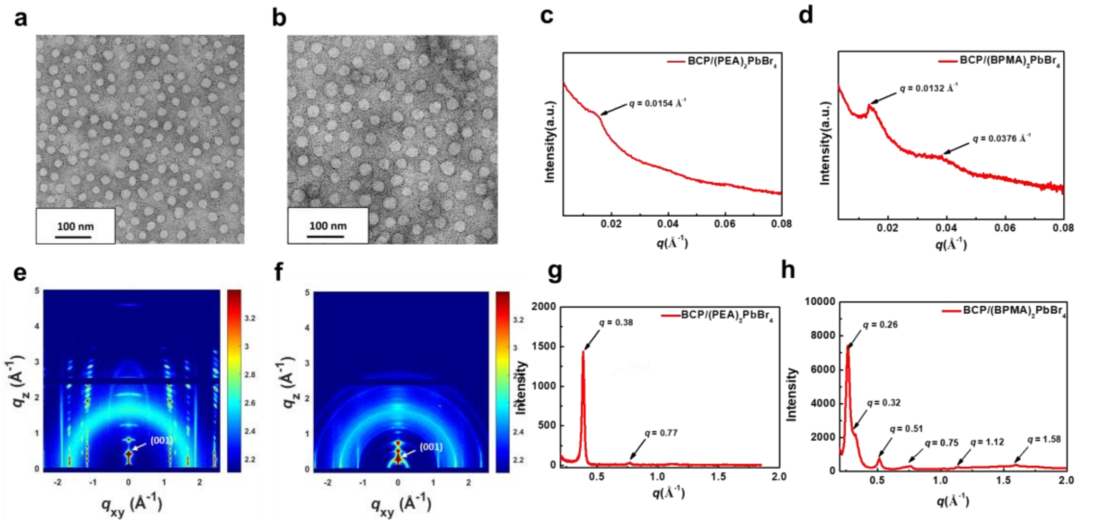

Figure S1. TEM image of (a)BCP/(PEA)$_2$PbBr$_4$ and (b)BCP/(BPMA)$_2$PbBr$_4$ composite film. GISAXS 1D profile of **(c)**BCP/(PEA)$_2$PbBr$_4$ and (d)BCP/(BPMA)$_2$PbBr$_4$ composite film. 2D diffraction pattern of **(e)**BCP/(PEA)$_2$PbBr$_4$ and (f)BCP/(BPMA)$_2$PbBr$_4$ composite film. 1D profile of scattering vector in out-of-plane direction of **(g)**BCP/(PEA)$_2$PbBr$_4$ and (h)BCP/(BPMA)$_2$PbBr$_4$ composite film.

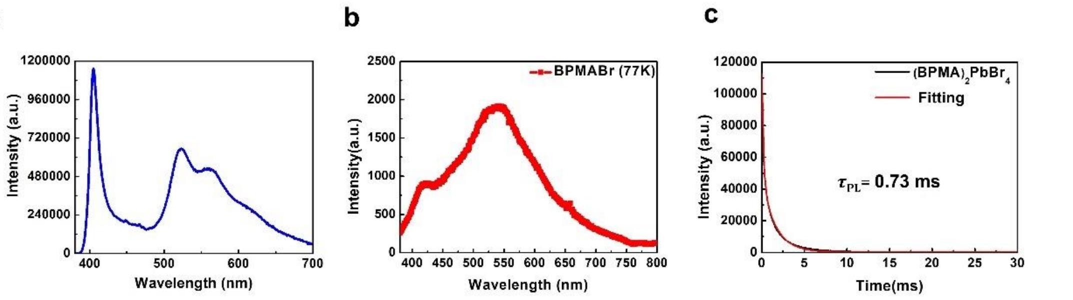

Figure S2. (a) Photoluminescence spectrum of (BPMA)$_2$PbBr$_4$ thin film with excitation wavelength of 365 nm. (b)Photoluminescence spectrum of BPMABr at 77 K with excitation wavelength of 325 nm. (c) TRPL decay with the fitting curves of (BPMA)$_2$PbBr$_4$ thin film at 530 nm with excitation wavelength of 375 nm.

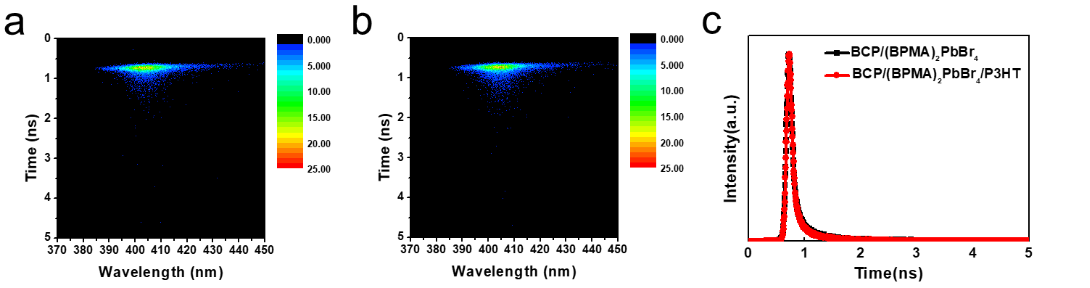

Figure S3. 2D contour plot of TRPL of (a) BCP/(BPMA)$_2$PbBr$_4$ and (b) BCP/(BPMA)$_2$PbBr$_4$/P3HT with excitation wavelength of 375 nm. TRPL decay curves with the fitting curves of (c) BCP/(BPMA)$_2$PbBr$_4$ and BCP/(BPMA)$_2$PbBr$_4$/P3HT.

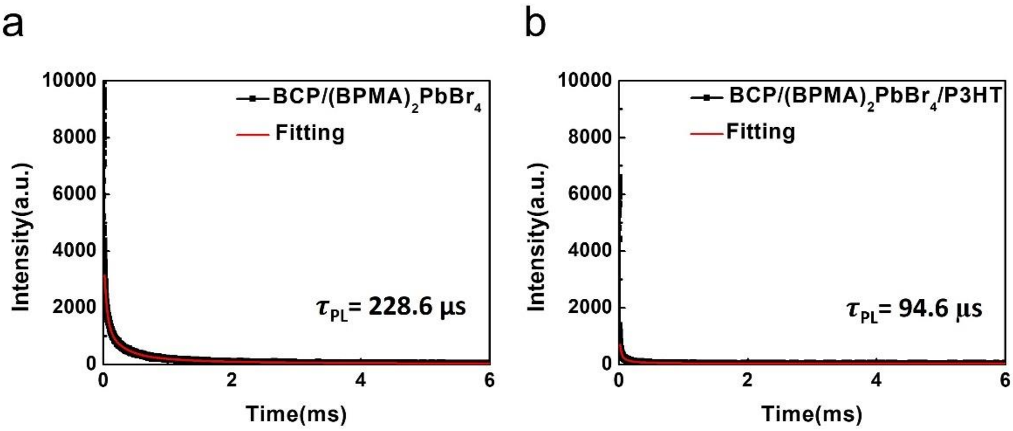

Figure S4. TRPL decay curves at the phosphorescence wavelength (527 nm) of (a) BCP/$(BPMA)_2PbBr_4$ and (b) BCP/$(BPMA)_2PbBr_4$/P3HT with excitation wavelength of 375 nm.

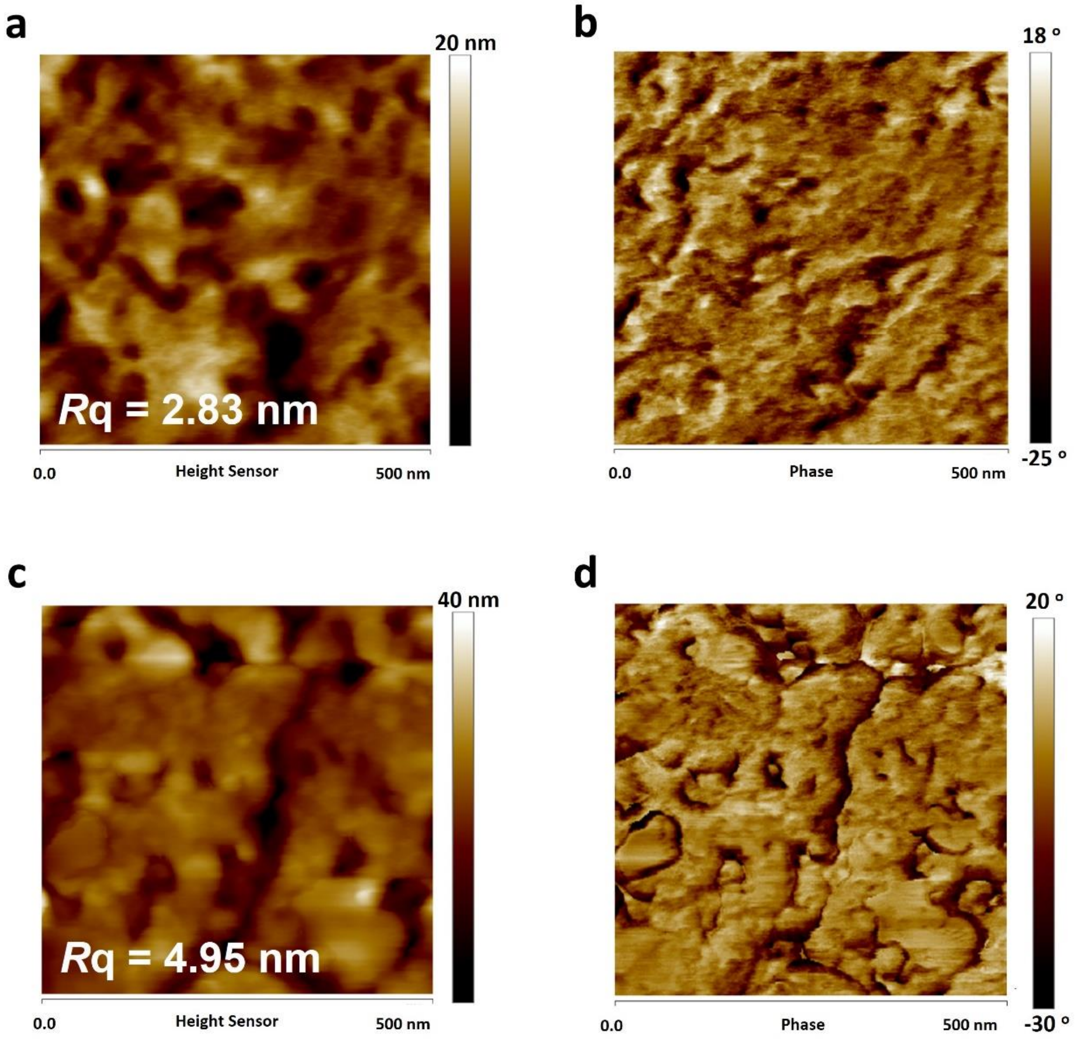

Figure S5. Atomic force microscope image of P3HT thin film on $(BPAM)_2PbBr_4$ and $(PEA)_2PbBr_4$. (a)Hight image and (b) phase image of P3HT/$(BPAM)_2PbBr_4$. (c)Hight image and (d) phase image of P3HT/$(PEA)_2PbBr_4$.

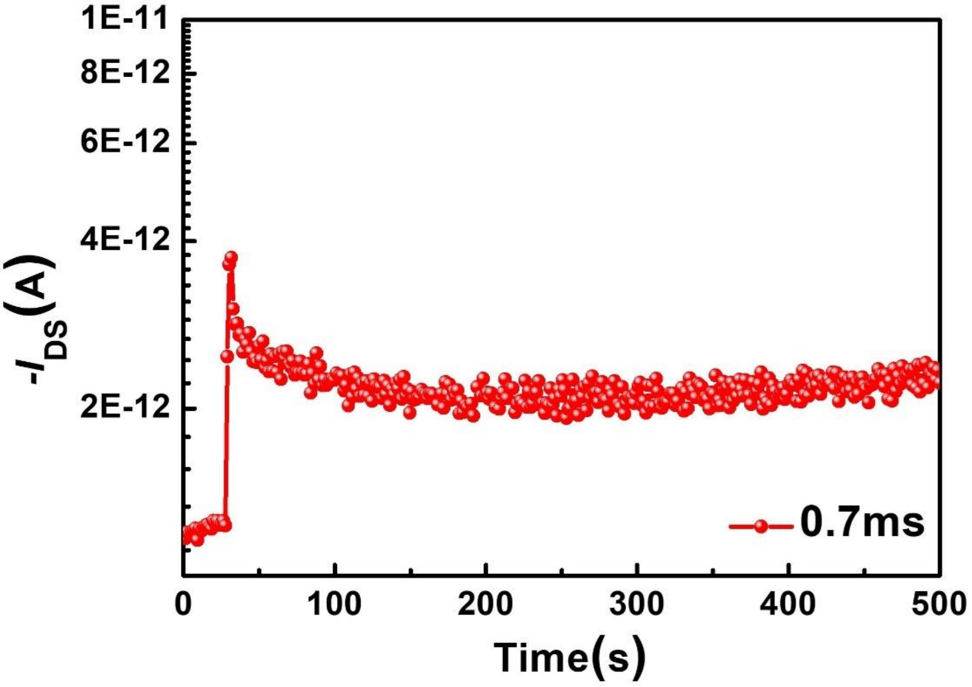

Figure S6. Temporal I_{DS} curves of the BCP/$(BPMA)_2PbBr_4$ based-photomemory at V_{DS} = −60 V with illuminating time of 0.7 ms (405 nm, 194 mW cm^{-2}).

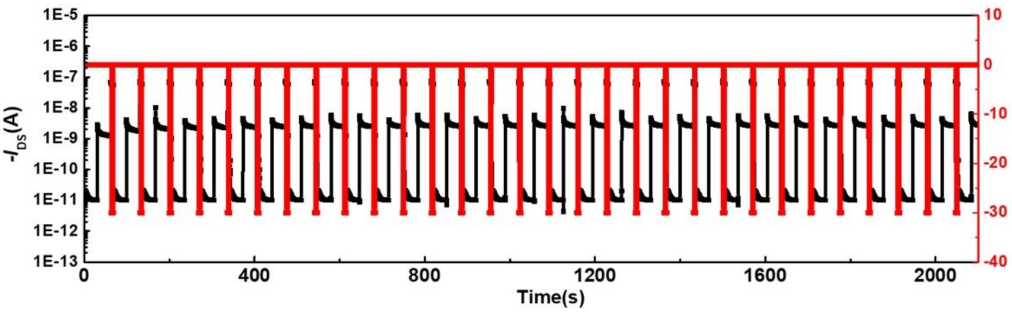

Figure S7. WRER switching test of the BCP/$(BPMA)_2PbBr_4$-based photomemory for 30 cycles which was measured at a fixed V_{DS} = −0.5 V and programmed by light illumination (405 nm, 194 mWcm^{-2}) for 1s and erased at V_{GS}= −30 V for 10 s.
